# Correction: Multisite surveillance for influenza and other respiratory viruses in India: 2016–2018

**DOI:** 10.1371/journal.pgph.0004140

**Published:** 2024-12-30

**Authors:** The PLOS GLOBAL PUBLIC HEALTH Staff

The eighth author’s name is written incorrectly. The correct name is: Mamta Chawla Sarkar.

The publisher apologizes for the error.
