# Supplementary material for: Multisite surveillance for influenza and other respiratory viruses in India: 2016–2018
Source: PLOS Glob Public Health. 2022 Nov 4;2(11):e0001001. doi: 10.1371/journal.pgph.0001001 (PMC10022084; doi:10.1371/journal.pgph.0001001)
Supplement: S1 Table — (DOCX) [file pgph.0001001.s001.docx]

**S1 Table: Monthly virus percent positivity by site, India (September 2016 to December 2018)**

- Coloured cells indicates months with percent positivity higher than overall percent positivity
