# Supplementary material for: Multisite surveillance for influenza and other respiratory viruses in India: 2016–2018
Source: PLOS Glob Public Health. 2022 Nov 4;2(11):e0001001. doi: 10.1371/journal.pgph.0001001 (PMC10022084; doi:10.1371/journal.pgph.0001001)
Supplement: Attachment — Submitted filename: Response to Reviewers.docx [file pgph.0001001.s003.docx]

From,

Dr Varsha Potdar

Lead, National Influenza center

National Institute of Virology, Pune, India

To,

The Editor

PLOS Global Public Health

Sir/Madam,

I would like to thank you and the reviewers for reviewing the manuscript and providing valuable suggestions. We have tried to address the suggestions. Following are the suggestions and changes made.

| Reviewer suggestion | Modification in article |
| --- | --- |
| To detail the basic strategies used to identify these cases at the sites and some idea of average daily recruitment | Trained staff visited outpatients clinics and inpatient wards on fixed day of week. Patients fulfilling the case definitions and consenting to participate were recruited in the study. Every week first 5-10 ARI/ SARI cases identified in outpatient clinics and inpatient wards of each center were enrolled into the study. |
| In Tables 1 & 2, for the OR estimation for different symptoms, it is not clear which is the reference category. | Reference group is added for each symptom |
| The font appears inconsistent between the main text and references. Also, spacing in the funding and acknowledgment section appears single and different from the sections above | The spacing and font type is made uniform. |
| To discuss site-specific differences for different viruses. | The variation in viral detection across centers could be due to difference in proportion of cases below 5 and above 60 years in different centers. Centers with lower proportion of under 5 years was (Chennai and Pune) or had higher proportion of adults>=60 years (Srinagar) had the lower detection of viruses in ARI and SARI cases |

I request you to consider the manuscript for publication

Thanking you

Yours Truly

Dr Varsha Potdar
